# Septal protein SepJ from the heterocyst‐forming cyanobacterium *Anabaena* forms multimers and interacts with peptidoglycan

**DOI:** 10.1002/2211-5463.12280

**Published:** 2017-08-30

**Authors:** Félix Ramos‐León, Vicente Mariscal, Natalia Battchikova, Eva‐Mari Aro, Enrique Flores

**Affiliations:** ^1^ Instituto de Bioquímica Vegetal y Fotosíntesis CSIC Universidad de Sevilla Spain; ^2^ Laboratory of Molecular Plant Biology Department of Biochemistry University of Turku Finland

**Keywords:** *Anabaena*, cyanobacteria, septal junctions

## Abstract

Heterocyst‐forming cyanobacteria grow as filaments that can be hundreds of cells long. Proteinaceous septal junctions provide cell–cell binding and communication functions in the filament. In *Anabaena* sp. strain PCC 7120, the SepJ protein is important for the formation of septal junctions. SepJ consists of integral membrane and extramembrane sections – the latter including linker and coiled‐coil domains. SepJ (predicted MW, 81.3 kDa) solubilized from *Anabaena* membranes was found in complexes of about 296–334 kDa, suggesting that SepJ forms multimeric complexes. We constructed an *Anabaena* strain producing a double‐tagged SepJ protein (SepJ‐GFP‐His_10_) and isolated the tagged protein by a two‐step affinity chromatography procedure. Analysis of the purified protein preparation provided no indication of the presence of specific SepJ partners, but suggested that SepJ is processed to remove an N‐terminal fragment. Additionally, pull‐down experiments showed that His_6_‐tagged versions of SepJ and of the SepJ coiled‐coil domain interact with *Anabaena* peptidoglycan (PG). Our results indicate that SepJ forms multimers, that it interacts with PG, and that the coiled‐coil domain is involved in this interaction. These observations support the idea that SepJ is a component of the septal junctions that join the cells in the *Anabaena* filament.

**Abbreviations**
BN: blue native
DDM: *n*‐dodecyl β‐d‐maltoside
HRP: horseradish peroxidase
PG: peptidoglycan
PVDF: poly(vinylidene difluoride)
SepJ‐CC: SepJ coiled‐coil domain

Heterocyst‐forming cyanobacteria are multicellular organisms that comprise cells performing oxygenic photosynthesis and CO_2_ fixation (vegetative cells) and N_2_‐fixing cells (heterocysts), which exchange nutrients and regulators 1. The cyanobacteria bear a Gram‐negative type of cell envelope, with a peptidoglycan (PG) layer and an outer membrane laying outside of the cytoplasmic membrane 2. The filaments of heterocyst‐forming cyanobacteria can be hundreds of cells long, and they consist of individual cells surrounded by their PG layer(s) but sharing the outer membrane 3. The PG layers of two adjacent cells appear to be fused in at least a fraction of the intercellular septa of the filament, which permits the isolation of murein sacculi corresponding to several cell units 4, 5. The cells in the filament are connected by proteinaceous structures termed septal junctions 6, 7, 8 (previously known as microplasmodesmata 9 or septosomes 10), which traverse septal PG through perforations that have been named nanopores 4. Structures described as ‘channels’, which have been observed by electron tomography 11, 12, likely correspond to the nanopores.

Proteins that may be components of septal junctions have been identified in the model heterocyst‐forming cyanobacterium *Anabaena* sp. strain PCC 7120 (hereafter *Anabaena*). SepJ (also known as FraG 13), FraC, and FraD are cytoplasmic membrane proteins that are located at the cell poles in the intercellular septa of the filament 14, 15. Inactivation of the corresponding genes results in a filament fragmentation phenotype, which is strongest in the case of *sepJ* 13, 14, 15. Although both SepJ and the FraCD proteins are required in *Anabaena* to produce a normal number of nanopores 16, there is evidence for the existence of at least two types of septal junctions, those related to SepJ and those related to FraCD 17.

In *Anabaena*, SepJ is a 751‐amino acid protein that contains an integral membrane domain (amino acids 412–751) and an extramembrane section (amino acids 1–411) 14. The latter consists of (a) 27 N‐terminal amino acids that are very conserved in SepJ from heterocyst‐forming cyanobacteria, (b) a coiled‐coil domain comprising two coiled‐coil motifs (residues 28–95 and 127–207), also strongly conserved, and (c) a linker domain (residues 208–401) which is not conserved in sequence but shares the amino acid composition being rich in Pro, Ser, and Thr residues 1. Functional GFP fusions 14 and bacterial adenylate cyclase two‐hybrid (BACTH) analysis 18, 19 indicated that the C terminus of SepJ is cytoplasmic. According to topology predictions, the extramembrane domain could be located either in the periplasm or in the cytoplasm 1, 8. The available experimental evidence, including immunolocalization of the coiled‐coil domain in the septa between adjacent cells 12 and interaction of SepJ with periplasmic proteins 18, 19, supports a periplasmic location of the long N‐terminal extramembrane section of SepJ. Such periplasmic location has been questioned, however, because of lack of an evidently predicted signal peptide in SepJ 12.

SepJ could contribute to the formation of septal junctions making protein complexes, and BACTH analysis has shown self‐interactions of SepJ when expressed in *Escherichia coli*, which suggests that SepJ can oligomerize 18. No direct evidence for complex formation involving SepJ has, however, been previously reported. To study whether SepJ forms complexes in its natural setting, in this work we addressed the isolation of SepJ from *Anabaena*. Additionally, to contribute to the understanding of the topology of SepJ, we investigated its possible interaction with PG.

## Materials and methods

### *Anabaena* strains and growth conditions

*Anabaena* sp. strains PCC 7120 (wild‐type), CSAM137 (*sepJ‐gfp‐*mut2) 14, and CSVM135 (*sepJ‐gfp‐*mut2‐His_10_) were used in this work. The *Anabaena* strains were grown in BG11 medium modified to contain ferric citrate instead of ferric ammonium citrate 20, or in BG11_0_ medium (BG11 further modified by omission of NaNO_3_), at 30 °C in the light (ca. 25–30 μmol photons·m^−2^·s^−1^), in shaken (100 r.p.m.) liquid cultures, or on plates with media solidified with 1% (w/v) Difco Bacto agar. For growth of the mutants, media were supplemented with streptomycin sulfate (Sm) and spectinomycin dihydrochloride pentahydrate (Sp), 2–5 μg·mL^−1^ each in liquid cultures and 5–10 μg·mL^−1^ in solid media. For the isolation of proteins from *Anabaena*, cultures were grown in BG11 medium supplemented with 10 mm NaHCO_3_ (BG11_C_). Either cultures bubbled with 1% CO_2_‐enriched air (method 1) or cultures grown in a chamber containing air enriched with 3% CO_2_ (method 2) were used. For the isolation of PG from *Anabaena*, 2‐L cultures were grown in BG11_C_ medium bubbled with 1% CO_2_‐enriched air. Bubbled cultures were illuminated laterally (50 μmol photons·m^−2^·s^−1^).

To construct strain CSVM135, the pCSVM135 plasmid was prepared encoding the SepJ‐GFP protein with the 10‐His tag at the C terminus. For that purpose, pCSAM135 that bears an altered *sepJ* gene, with the *gfp‐mut2* gene substituting for the last 27 bp of *sepJ* 14, was used as a template. A DNA fragment was amplified from pCSAM135 with primers gfp‐His1 (5′ GTCCT GCAGT TAATG ATGAT GATGA TGATG ATGAT GATGA TGGAT ATCTT TGTAT AGTTC ATCCA T) and Fw (5′ GTAAA ACGAC GGCCA GT). The amplified fragment (which contains a 515‐bp DNA fragment from the 3′‐terminal portion of *sepJ*, the *gfp‐*mut2 gene, and a 3′‐terminal sequence encoding the His tag) was inserted as a *Pst*I fragment into pCSV3 21 producing pCSVM135. This plasmid was transferred to wild‐type *Anabaena* by conjugation, which should have integrated the construct through single recombination at *sepJ* (see Fig. 2A below), and exconjugants were isolated as Sm^R^ Sp^R^ clones. The genetic structure of strain CSVM135 in the *sepJ* region was corroborated by PCR using primers gfp‐6 (5′ GGATC CAGTA AAGGA GAAGA AC) and Alr2338‐2 (5′ TTTTC TGTGG TGAGG TGC). For staining of heterocyst polysaccharide layers, cell suspensions were mixed (1 : 2) with a filtered 1% (w/v) Alcian blue (Sigma, St. Louis, MO, USA) solution in water and visualized by standard light microscopy. The percentage of heterocysts was determined by counting a total of 1755 cells in filaments.

### Isolation of SepJ complexes from *Anabaena*

Membranes were isolated from *Anabaena* by two different methods. In the first method, filaments from 1‐L cultures (BG11_C_ medium) were collected by filtration, washed twice with PBS buffer (5.5 mm Na_2_HPO_4_, 1.8 mm KH_2_PO_4_, 137 mm NaCl, 2.7 mm KCl) and resuspended in 10 mL of PBS buffer containing protease inhibitors. The cells were broken by passage through a French press three times at 20 000 psi. The resulting suspension was centrifuged at 7000 ***g*** for 20 min. The resulting supernatant was subjected to centrifugation at 150 000 ***g*** for 1 h, the pellet was washed twice with PBS buffer, and the isolated membranes were solubilized as explained below (Protein analysis). In the second method, filaments from 300‐mL cultures were harvested by centrifugation at 7000 ***g***, washed twice with the PBS buffer, and resuspended in 2 mL of the PBS buffer supplemented with 30 mm CaCl_2_, 800 mm sorbitol, and 1 mm ε‐amino‐*n*‐caproic acid. The cells were broken with glass beads by vortexing 6 × 1 min at 4 °C. After removing cell debris by low‐speed centrifugation, isolated membranes were collected by centrifugation at 18 000 ***g*** for 15 min, resuspended in PBS buffer supplemented with 1% *n*‐dodecyl β‐d‐maltoside (DDM), and 20 mm Pefabloc and incubated at 4 °C for 15 min. Nonsolubilized proteins were removed by centrifugation at 20 000 ***g*** for 20 min. The supernatant was used to purify the His‐tagged SepJ proteins on a His SpinTrap (GE Healthcare, Little Chalfont, UK) column following the instructions of the manufacturer. After loading, the resin was washed four times with PBS buffer supplemented with 1% DDM and 20 mm imidazole, and proteins bound to the resin were eluted with 200 μL of PBS buffer containing 1% DDM and 300 mm imidazole.

For further purification of SepJ complexes, the eluted fraction was diluted to 1 mL with PBS buffer containing 1% DDM and incubated with μMACS anti‐GFP MicroBeads (Miltenyi Biotec, Bergisch Gladbach, Germany) at 4 °C for 1 h. The sample and beads were loaded into a MACS column (Miltenyi Biotec), washed with 3 mL of PBS containing 1% DDM, and eluted with 100 μL of elution buffer [50 mm Tris/HCl (pH 6.8), 50 mm DTT, 1% SDS, 1 mm EDTA, 0.0005% bromophenol blue, and 10% glycerol]. Eluted fractions were analyzed by SDS/PAGE. Bands were excised from SDS/PAGE, digested with trypsin, and analyzed by LC‐MS/MS as described in Battchikova *et al*. 22.

### Production of proteins in *Escherichia coli*

To produce the entire SepJ protein fused to the His_6_‐tag (SepJ‐His_6_) in *E. coli*, the *sepJ* gene was amplified using primers alr2338‐17 (5′ TCCAT GGGGC GATTG AGAAG CGA) and alr2338‐20 (5′ ACTCT CGAGA TTGGC AGGTT TGT). The PCR product was digested with *Nco*I and *Xho*I and cloned into pET28‐b (Novagen, Merck, Darmstadt, Germany), producing pCSVM100. The coiled‐coil domain of SepJ fused to a His6‐tag (SepJ‐CC‐His_6_) was encoded by pCSVM98 23. The *E. coli* BL21 strains carrying plasmids pCSVM98 or pCSVM100 were grown in 30 mL of LB medium containing kanamycin (50 μg·mL^−1^) and 2% glucose at 37 °C overnight. Cells were collected by centrifugation and resuspended in 1 mL of LB medium. About 500 μL of the cell suspension was used to inoculate 500 mL of LB medium supplemented with 50 μg kanamycin·mL^−1^. When absorbance at 600 nm reached 0.6, protein expression was induced by the addition of 1 mm of IPTG. After incubation at 37 °C for at least 2 h, cells were collected by centrifugation at 5000 ***g*** for 15 min, washed, and resuspended in 10 mL of PBS buffer supplemented with 5 μg·μL^−1^ DNase I and protease inhibitors. Cells were broken by passage through a French Pressure cell twice at 20 000 psi. The resulting suspension was centrifuged at 15 000 ***g*** for 10 min to remove cell debris. To isolate SepJ‐CC‐His_6_, the supernatant was supplemented with DDM at 1%. To isolate SepJ‐His_6_, the supernatant was subjected to centrifugation (150 000 ***g**,* 1 h, 4 °C) and the precipitated membranes were resuspended in 1 mL of PBS buffer containing 1% DDM. The protein preparations obtained were subjected to a His SpinTrap (GE Healthcare) column as described above. SepJ‐His_6_ and SepJ‐CC‐His_6_ proteins were eluted from the resin with 300 μL of the PBS buffer containing 1% DDM and 300 mm imidazole.

### Protein analysis

**Figure 1:**
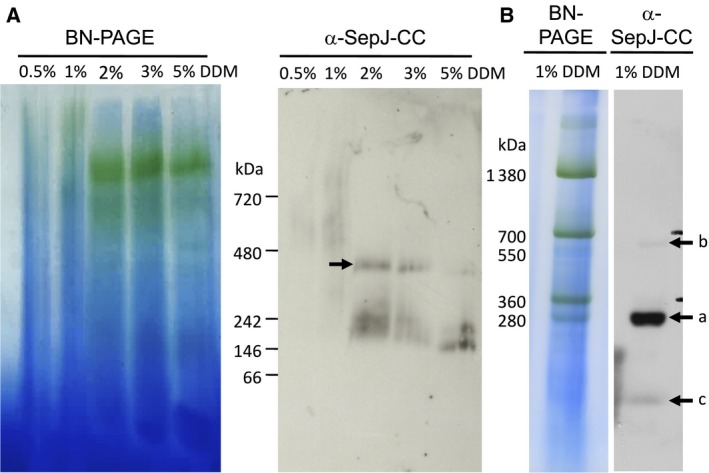
Isolation of SepJ complexes from *Anabaena* membranes. (A) Picture of a PAGE gel (left panel) and western blot analysis using anti‐SepJ‐CC antibodies of the same gel (right panel). Membranes were isolated by disrupting the cells in a French pressure cell, and the gel corresponds to a 4–16% polyacrylamide gradient. Arrow points to a SepJ‐containing complex. Size markers, NativeMark™ Unstained Protein Standard. (B) Picture of a PAGE gel (left panel) and western blot analysis using anti‐SepJ‐CC antibodies of the same gel (right panel). Membranes were isolated by disrupting the cells with glass beads, and the gel corresponds to a 4–10% polyacrylamide gradient. Arrows point to SepJ‐containing complexes. Size markers, photosynthetic complexes.

For blue native (BN)‐PAGE, *Anabaena* membranes containing 200 μg of total protein were collected by centrifugation at 20 000 ***g*** for 20 min. Then, 10 μL of resuspension buffer (25 mm Bis‐Tris, pH 7, 20% glycerol, 500 mm MgCl_2_, 20 mm Pefabloc) was added to the pellet and incubated on ice for 10 min. After that, 10 μL of resuspension buffer supplemented with DDM at the final concentration indicated in each case was added. Samples were incubated in ice for 10 min. Preparations were centrifuged at 18 000 ***g*** for 20 min, and the supernatant containing the solubilized membranes (about 10 μg·μL^−1^ final concentration) was transferred to a new tube. For experiments like that shown in Fig. 1A, native PAGE 4–16% 1.5‐mm 16 cm × 16 cm Bis‐Tris gels were prepared as described 24. For all other experiments, the BN gel was prepared as described 25 with some modifications 26, and the Hoefer SE 250 running system was used. Samples were supplemented with 4× BN buffer containing 100 mm Bis‐Tris (pH 7.0), 80% (w/v) glycerol, and 40 mm Pefabloc. Prior to loading, samples were supplemented with a one‐tenth volume of sample buffer containing 100 mm Bis‐Tris pH 7.0, 500 mm ε‐amino‐*n*‐caproic acid, 30% (w/v) sucrose, and 50 mg·mL^−1^ Serva Blue G. Samples were loaded on an equal protein basis of 150 μg per well in a 4–10% acrylamide gradient gel. After running, proteins were transferred to poly(vinylidene difluoride) (PVDF) membrane filters. Size standards in BN gels were NativeMark™ Unstained Protein Standard (from Life Technologies, Carlsbad, CA, USA) or photosynthetic complexes: PSI tetramer (high molecular PSI), 1380 kDa; PSI dimer, 700 kDa; PSII dimer, 550 kDa; PSI monomer, 360 kDa; and PSII monomer, 280 kDa. For molecular size estimation, logarithm of standards’ MW and their corresponding mobility were plotted to obtain straight lines, and their equations were used to predict the size of bands of interest.

For SDS/PAGE, the procedure of Laemmli 27 was followed as described 28. Samples were mixed with 1 volume of 2× sample buffer, incubated at 30 °C for 2 h, and run in a 12% Laemmli SDS/PAGE system. For the detection of *Anabaena* SepJ, proteins were transferred to PVDF membrane filters and immunoblot was carried out using an antibody against the SepJ coiled‐coil domain (diluted 1 : 500) as previously reported 23. Protein size standards were Bio Blue Prestained Ladder (gTPbio, C. Viral, Seville, Spain).

### Peptidoglycan isolation and binding assays

Murein sacculi (PG) were isolated as described by de Pedro *et al*. 29, with several modifications. Cells grown in 2 L of BG11_C_ medium (bubbled with 1% CO_2_‐enriched air) were collected by filtration and resuspended in 15 mL of PBS buffer. The cell suspension was concentrated to 50 μg chlorophyll *a*·mL^−1^ and 3 mL (containing cells corresponding to 150 μg chlorophyll *a*) was used for PG isolation. Filaments were fragmented by sonication in a bath to a homogenous suspension of single cells or two‐ to three‐cell‐long filaments followed by centrifugation at 3000 ***g*** for 10 min and resuspension of the cell pellet in 1 mL of the PBS buffer. Drops of the cell suspension were slowly added to 10 mL of boiling 6% (w/v) SDS solution. The boiling proceeded for 1 h, with strong stirring, followed by incubation at 37 °C overnight (gently stirred). Next, samples were boiled for 1 h, and PG was precipitated by centrifugation at 320 000 ***g***, 25 °C, 30 min. The PG pellet was resuspended in 3 mL of 3% (w/v) SDS, boiled for 2 h, and precipitated by centrifugation (320 000 ***g***, 25 °C, 30 min). The pellet was resuspended in 2 mL of 0.05% (w/v) SDS and boiled for 2 h. After centrifugation as above, the pellet was resuspended in 1.5 mL of 50 mm sodium phosphate buffer (pH 6.8) containing 50 μg (corresponding to 2 U) of α‐chymotrypsin (from bovine pancreas; Sigma). The suspension was incubated at 37 °C overnight. After adding 0.5 mL of water and 0.75 mL of 6% SDS, the samples were boiled for 2 h and then precipitated by centrifugation as above. In order to remove SDS, the pellet was washed three times by suspension in doubly distilled water and centrifugation at 320 000 ***g***, 25 °C, 15 min. Finally, the PG pellet was resuspended in 120 μL of water.

SepJ‐His_6_ and SepJ‐CC‐His_6_ purified as described earlier were investigated for the PG binding. Bovine serum albumin and horseradish peroxidase (HRP), both from Sigma‐Aldrich, were used as negative controls. A volume of protein preparation to give 5 μm final concentration of protein (in PBS buffer containing 1% DDM) and 30 μL of PG solution (OD_600_ 0.83) was mixed in a final volume of 50 μL. The mixture was incubated at room temperature for 1 h with gentle shaking, followed by centrifugation at 170 000 ***g*** for 30 min (25 °C). The pellet containing PG and the putative interacting proteins was resuspended in 50 μL of the PBS buffer containing 1% DDM, collected again by centrifugation as above, and resuspended in 50 μL of the same buffer. The protein content of the samples was quantified by the Lowry procedure, and the samples were subjected to SDS/PAGE and staining with Coomassie Blue G‐250. Identity of proteins was confirmed by MALDI‐TOF performed at Servicio de Proteómica, Instituto de Bioquímica Vegetal y Fotosíntesis (CSIC, Seville).

## Results

### SepJ multimers

Membranes were first isolated from *Anabaena* by disrupting the cells in a French pressure cell (see Materials and methods). The membranes were solubilized with 0.5–5% DDM, and the solubilized material was analyzed by BN‐PAGE and western blotting performed with antibodies raised against the coiled‐coil domain of SepJ (anti‐SepJ‐CC). This analysis showed that a complex that reacted with anti‐SepJ‐CC (arrow in Fig. 1A, right panel) was solubilized from the membranes by using 2% DDM, whereas lower DDM concentrations were insufficient to solubilize the complex and higher DDM concentrations apparently destabilized the complex releasing material of lower MW. In three independent isolations, the complex solubilized with 2% DDM had an apparent MW of 334 ± 10 kDa (mean ± SD). Nonetheless, as observed directly in the BN‐PAGE gels, the isolation of proteins from membranes by this method did not produce sharp bands as evidenced with the photosynthetic complexes (chlorophyll‐containing materials, green color, in Fig. 1A, left panel). We then isolated membranes after disrupting *Anabaena* with glass beads, and the membranes were solubilized with 1% DDM permitting the visualization of the photosynthetic complexes as sharp bands (Fig. 1B, left panel). Western blot analysis with anti‐SepJ‐CC showed the presence of a major band of about 296 kDa (arrow a in Fig. 1B, right panel) and minor bands of about 600 kDa (arrow b) and 141 kDa (arrow c). Sharp bands were not observed when 3% DDM was used, suggesting that complexes were destabilized (not shown). The SepJ protein is 81.3 kDa in size, and therefore, the anti‐SepJ‐CC material observed after solubilizing the membranes isolated by neither of the methods used could be directly interpreted as corresponding to a complex containing an integer of SepJ subunits. However, although the complexes might contain additional proteins, inaccurate determination of their MW would also be possible because of different mobility as compared to the size markers used, which were a set of soluble proteins in Fig. 1A and photosynthetic complexes in Fig. 1B. The major bands observed could therefore correspond to a tetramer of SepJ proteins (expected MW, 325 kDa).

### Production and purification of double‐tagged SepJ

**Figure 2:**
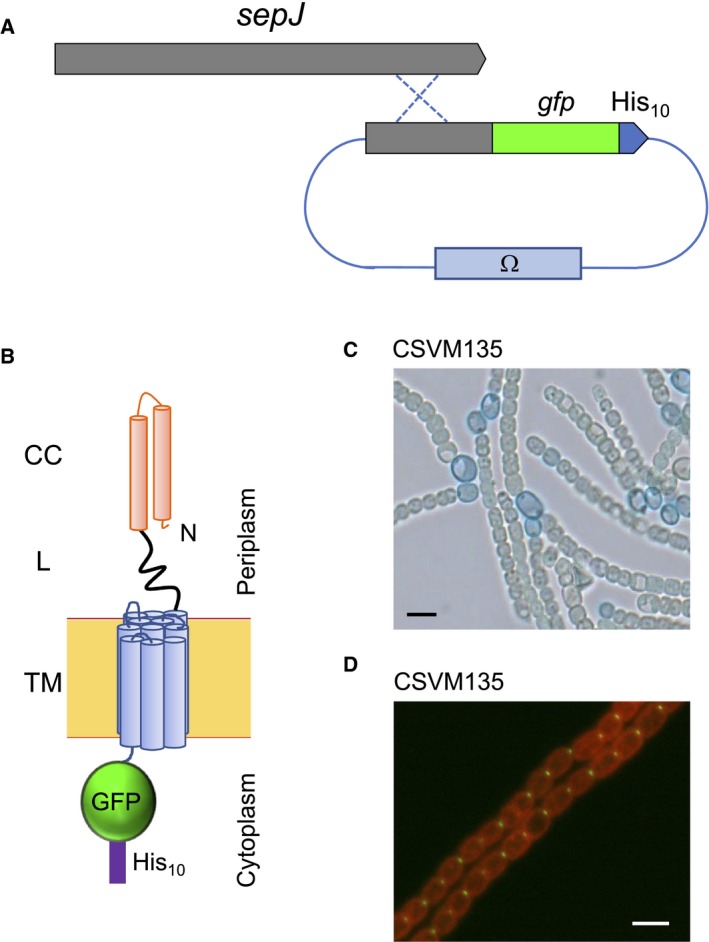
*Anabaena* strain expressing a double‐tagged SepJ protein. (A) Schematic of the genetic strategy used to generate strain CSVM135. Ω, gene cassette encoding Sm^R^ Sp^R^. (B) Schematic of the double‐tagged SepJ protein produced in strain CSMV135 including its predicted localization in the cytoplasmic membrane. The GFP protein is fused to the SepJ C terminus, and a His_10_‐tag is in turn fused to the GFP. CC, coiled‐coil domain; L, linker domain; TM, transmembrane domain. (C) Filaments of strain CSVM135. Heterocysts were stained with Alcian blue, which highlights in blue color the polysaccharide layer of the heterocyst envelope. Size bar, 5 μm. (D) Micrograph showing the localization of SepJ‐GFP‐His_10_ by fluorescence microscopy. The micrograph is an overlay of the GFP fluorescence (green) and cyanobacterial autofluorescence (red). The fusion protein is correctly focused at the cell poles in the intercellular septa. Size bar, 5 μm.

**Figure 3:**
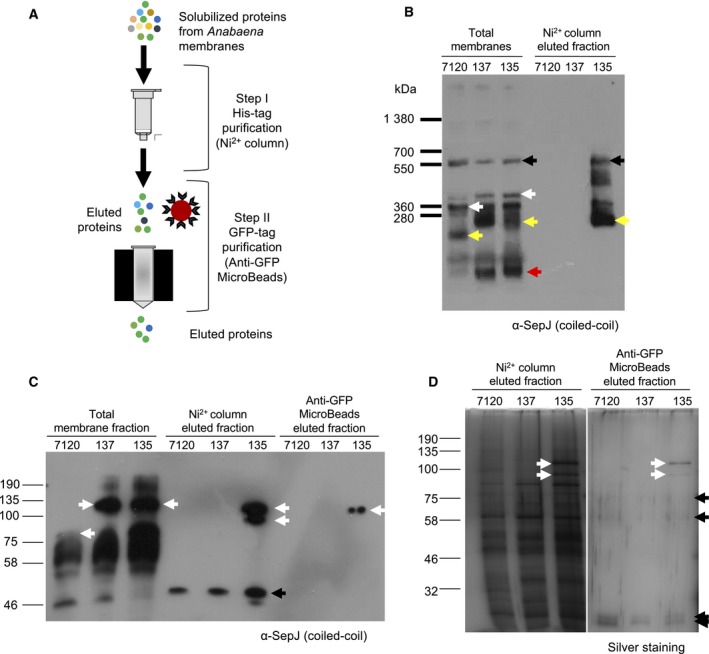
Isolation and analysis of SepJ complexes from *Anabaena* membranes using a double‐tagged SepJ protein. (A) Schematics of the procedure. After solubilization of membranes from *Anabaena*, the proteins were incubated with a nickel resin (step I) and eluted using imidazole. The resulting eluate was incubated with anti‐GFP microbeads (step II), represented as a red circle surrounded by antibodies. The mixture was then loaded into a MACS column where microbeads were retained together with GFP‐tagged proteins bound to the antibodies. Elution from the MACS column was carried out using denaturing conditions. (B) Membrane fraction and material eluted from step I were subjected to BN‐PAGE and western blot analysis using anti‐SepJ‐CC antibodies. High MW reacting material was found in the membrane preparations from the three strains and in material eluted only from strain CSVM135. Black arrows point to a possible unspecific band, white arrows point to SepJ complexes that could correspond to those observed in Fig. 1 for the wild‐type and of increased size in strains producing tagged SepJ proteins, yellow arrows point to anti‐SepJ‐CC‐reacting material that is of increased size in the strains producing tagged SepJ proteins as compared to the wild‐type, and a red arrow points to putative SepJ monomers in the strains producing tagged SepJ proteins. (C) Western blot analysis using anti‐SepJ‐CC antibodies after SDS/PAGE of samples from the membrane preparations and steps I and II. White arrows point to possible SepJ bands in the membrane preparations and eluates. Black arrow points to an unspecific band. (D) Proteins contained in eluted fractions (steps I and II) were separated by SDS/PAGE and silver‐stained. White arrows point to two putative forms of SepJ‐GFP‐His_10_, and black arrows point to unspecific proteins present in eluted fractions from the three strains. 7120, material from wild‐type strain PCC 7120; 137, from strain CSVM137 producing SepJ‐GFP; 135, from strain CSVM135 producing SepJ‐GFP‐His_10_.

To purify SepJ, we constructed an *Anabaena* strain, CSVM135, expressing a SepJ version C terminally fused to a double tag (SepJ‐GFP‐His_10_; Fig. 2B). Because in *sepJ* mutants, heterocyst formation is arrested early in the differentiation process 13, 14, the functionality of SepJ‐GPF‐His_10_ was assessed by testing the capability of heterocyst differentiation in strain CSVM135. Filaments of the CSVM135 strain were incubated in medium lacking combined nitrogen for 48 h and subjected to staining with Alcian blue, which is useful to detect bacterial polysaccharides 30 and, hence, the polysaccharide layer in the heterocyst envelope 31. Microscopic inspection of filaments showed the presence of heterocysts (Fig. 2C), which represented 8.66% of the total cells in the filaments, a figure similar to that usually found in wild‐type *Anabaena* (7–10%). Fluorescence microscopy of BG11‐grown filaments of the CSVM135 strain showed that the SepJ‐GPF‐His_10_ protein was correctly located at the middle of the intercellular septa (Fig. 2D). Thus, this strain was suitable for SepJ purification and analysis. The wild‐type *Anabaena* and the CSAM137 strain expressing a SepJ‐GFP were used as controls. The predicted MW values for the SepJ monomers produced in these strains are 81.3 kDa for SepJ from the wild‐type, 107.5 kDa for SepJ‐GFP from CSAM137, and 108.8 kDa for SepJ‐GFP‐His_10_ from CSVM135. The two‐step procedure that was set up to isolate SepJ attempting to preserve possible SepJ‐containing complexes is summarized in Fig. 3A.

**Figure 4:**
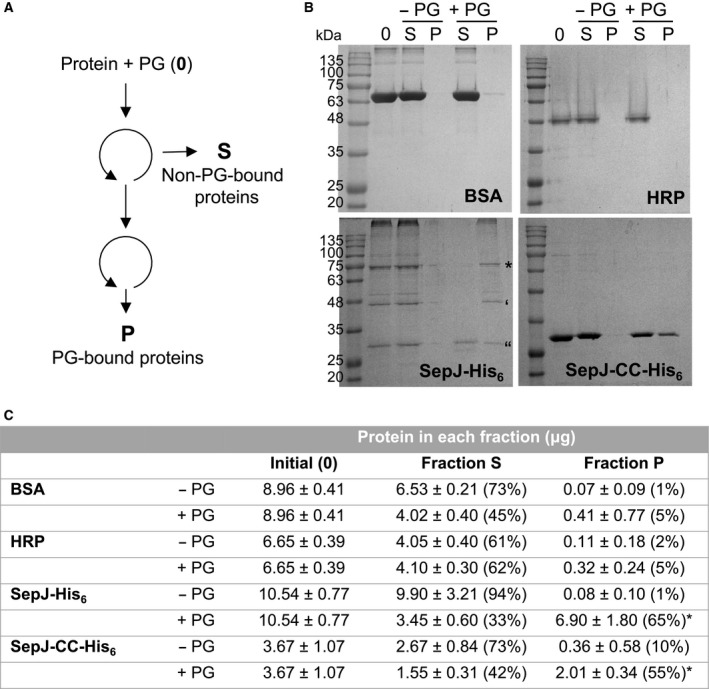
Analysis of the SepJ–PG interaction. (A) Schematic of the procedure used. PG and the indicated protein were mixed and centrifuged, and the precipitate was resuspended and centrifuged again as described in Materials and methods. (B) The first supernatant (S), where proteins unbound to PG remain, and the second precipitate (P), containing PG and bound proteins, were subjected to SDS/PAGE and stained. The first lane in each gel (0) contains a sample of the protein added to the incubation. In the SepJ‐His_6_ SDS/PAGE gel: *, full‐length SepJ‐His_6_ protein (determined MW, 79.4 kDa; expected MW, 82 kDa); ‘ and “, degradation products of estimated 50.9 and 30.9 kDa, respectively, that together make a full‐length SepJ‐His_6_ protein. MALDI‐TOF analysis showed that the 30.9‐kDa band corresponds to an N‐terminal fragment of SepJ. (C) Quantification of protein in each fraction after the PG‐binding assay (mean ± SD, *n* = 3). Samples containing only isolated PG were also quantified and the amount found (equivalent to 7.33 ± 0.44 μg of protein) was subtracted from those fractions that contained PG. The percentage of protein is presented relative to the amount added to the assay. Asterisks indicate those conditions in which the relative amount of protein was significantly different from that found in the precipitated BSA control (assessed by Student's *t*‐test, *P* < 0.05).

Total membrane fractions from *Anabaena* filaments were obtained by disrupting the cells with glass beads. The membrane proteins were solubilized with 1% DDM and subjected to BN‐PAGE (Fig. 3B) and SDS/PAGE (Fig. 3C). SepJ protein variants were detected by western blot analysis performed with anti‐SepJ‐CC. After BN‐PAGE, several bands reacting with the antibodies were observed in the membrane fractions from the three strains. For the largest SepJ assemblies, apparent MW values were in the range of 610–655 kDa, which are about the size of hexamers in the strains producing tagged SepJ proteins. However, because these bands were of a similar size in the three strains and the size of hexamers should be clearly different for wild‐type SepJ (488 kDa) and SepJ from CSAM137 (645 kDa) and CSVM135 (653 kDa), they may correspond to unspecific antibody‐reacting materials. We therefore looked at bands that were increased in size in the strains producing the SepJ‐GFP and SepJ‐GFP‐His_10_ fusions as compared to the wild‐type. A band that could correspond to the complexes noted in Fig. 1 was observed in the wild‐type preparation (322 kDa) and possible corresponding bands with an increased size were observed in the strains producing tagged SepJ (about 387 and 392 kDa for CSAM137 and CSVM135, respectively; Fig. 3B, white arrows). Another major band of about 207 kDa was observed in the wild‐type, and possible corresponding bands of about 260 kDa were observed in the strains producing tagged SepJ (Fig. 3B, yellow arrows). Because of possible altered mobility of the protein complexes, and because SepJ is a multidomain protein that may be easily degraded (see SepJ in Fig. 4) and appears to be processed (see description of Fig. 3D below), these bands are difficult to assign to specific multimers; additionally, the presence of other protein(s) in the SepJ complexes is possible. Finally, a putative tagged SepJ monomer was observed in strains CSAM137 (115 kDa) and CSVM135 (118 kDa; Fig. 1B, red arrow). On the other hand, SDS/PAGE demonstrated occurrence of SepJ monomers in the three investigated strains (white arrows in total membrane fractions; Fig. 3C), but putative SepJ degradation or processed products were also observed.

Protein preparations from the three strains were subjected to Ni^2+^ affinity purification (Fig. 3A), and the eluates were analyzed by BN‐PAGE and SDS/PAGE and western blotting. As expected, anti‐SepJ‐CC‐reacting proteins were observed only in the material from the CSVM135 strain encoding the SepJ‐GFP‐His_10_ protein (Fig. 3B,C). In BN‐PAGE, in addition to the 655‐kDa band that may be unspecific, materials reacting with anti‐SepJ‐CC were observed including a major 260‐kDa band (yellow arrow in Fig. 3B). As described above, these bands are difficult to assign to specific SepJ complexes. SDS/PAGE of the purified protein preparation from strain CSVM135 revealed two bands reacting with anti‐SepJ‐CC (Fig. 3C, Ni^2+^ eluted fraction, white arrows) that likely correspond to two forms of SepJ as discussed below. A low‐MW band was also detected that, because it was also observed in the material from the control strains, should correspond to a protein reacting nonspecifically with anti‐SepJ‐CC. Visualization by silver staining showed the presence of numerous proteins in material from the three strains eluted from the nickel resin, but the two SepJ forms were evident in the eluate from strain CSVM135 (Fig. 3D).

After the presence of SepJ in the eluate from the nickel resin was confirmed by SDS/PAGE, the preparations were further incubated with anti‐GFP MicroBeads and passed through a MACS column. The materials eluted from this column were subjected to SDS/PAGE and visualized by western analysis with anti‐SepJ‐CC and by silver staining. A band reacting with the antibodies was observed only in material that originated from strain CSVM135 (SepJ‐GFP‐His_10_) (Fig. 3C). Silver staining revealed six bands in this preparation. Four of them (black arrows) were also detected in material from the negative controls (Fig. 3D) and thus corresponded to nonspecific contaminants. The two bands unique for the CSVM135 strain (indicated by white arrows) were analyzed by mass spectrometry (see Appendix S1). Peptides that originated from SepJ and GFP were detected in both samples, confirming that these two bands correspond to SepJ‐GFP‐His_10_. Whereas the estimated size of the upper band, 109 kDa, corresponded to the full SepJ‐GFP‐His_10_ protein, the size of the lower band, 98 kDa, could correspond to a degradation or processed product of SepJ‐GFP‐His_10_. Because peptides of the GFP (fused to the C‐terminal part of the protein) were detected by mass spectrometry, the missing fragment should correspond to the N‐terminal part of the protein. Indeed, whereas peptides starting at amino acid residue 43 of SepJ were detected in both bands, a peptide corresponding to residues 4–13 was detected only in the upper band. The truncated protein (lower band) would retain most of the coiled‐coil domain as well as the GFP and His_10_ tags. Therefore, it could be copurified with full‐length SepJ‐GFP‐His_10_ during the two affinity chromatography steps and could still be recognized by anti‐SepJ‐CC as observed in Fig. 3C (fraction eluted from the nickel resin; not observed in the fraction eluted from the MACS column perhaps because of its low abundance as shown by silver staining in Fig. 3D).

### Interaction of SepJ with peptidoglycan

As summarized in the Introduction, septal junctions are thought to traverse the septal PG layers through nanopores. If SepJ is a component of septal junctions and the long extramembrane section of SepJ is periplasmic, SepJ could interact with PG. To test this possibility, the entire SepJ protein and its coiled‐coil domain (SepJ‐CC) were produced in *E. coli* as His_6_‐tagged proteins and purified. In parallel, murein sacculi (which are made of PG) were isolated from *Anabaena* (see Materials and methods). The protocol summarized in Fig. 4A was followed using as negative controls BSA and HRP. The same amount of each protein (in molar terms) was incubated with a fixed amount of PG as described in Materials and methods. Samples were then subjected to ultracentrifugation, and the precipitate, containing PG and putative interacting proteins, was washed to minimize proteins retained by nonspecific interactions. Samples were then analyzed by SDS/PAGE (Fig. 4B), and the protein content of the PG precipitates was quantified. Both the entire SepJ protein and the coiled‐coil domain were present in the precipitated fraction of PG. Controls without PG, or with BSA or HRP instead of SepJ, showed absent, or negligible, protein precipitation (Fig. 4B).

Quantification of protein in the samples subjected to SDS/PAGE is shown in Fig. 4C. The amounts of protein in fraction S, corresponding to unbound proteins, and in fraction P, corresponding to PG‐bound proteins, were determined by the Lowry assay. Because PG reacts giving increased absorbance, figures were corrected for the presence of PG. A significant percentage of SepJ‐His_6_ (about 65%) was bound to the PG, and the ability of binding PG was also observed for the isolated coiled‐coil domain (SepJ‐CC‐His_6_), as 55% of the added amount was recovered with the PG. A very small percentage of the control proteins (5%) precipitated with PG, indicating that the interaction of PG with SepJ and the coiled‐coil domain was specific. These results show that SepJ and its coiled‐coil domain can interact with PG.

## Discussion

The SepJ protein is needed for the formation of septal junctions in *Anabaena*, and the septal junctions likely traverse the septal PG through nanopores 16. It has been shown that the septal junctions may be 12–26 nm in length and 5.5–14 nm in diameter, which would fit well into the nanopores that have a diameter of 15–20 nm 1. Because of the large size of septal junctions, the proteins constituting these structures can be predicted to make large complexes. FraC and FraD are also necessary to make a normal number of nanopores in *Anabaena* 16, but there is currently no evidence for the formation of SepJ–FraCD complexes. Data presented in this work show that SepJ isolated from *Anabaena* membranes can be mainly found in complexes of a size ranging from 296 to 334 kDa depending on the method of extraction and analysis. On the other hand, analysis of an isolated doubly tagged SepJ‐GFP‐His_10_ preparation gave no indication of the presence in the complexes of proteins other than SepJ. Therefore, although the presence in SepJ‐related septal junctions of protein constituents additional to SepJ cannot be ruled out, our results are consistent with the formation in *Anabaena* of SepJ multimers that could include tetramers (predicted MW, 325 kDa).

SepJ has previously been shown to interact with cell division protein FtsQ 18 and with the novel PG‐binding protein SjcF1 19, but these proteins were not found in the SepJ complexes studied in this work. The interactions of SepJ with these proteins can be thought as transient. FtsQ is a component of the divisome, which directs SepJ to the cell poles 18, but once SepJ is in its final location no further interaction with the divisome is expected. SjcF1 appears to be a regulator of the size of the nanopores 19, but once the nanopores have been formed and the septal junction is in its location, no interaction between SepJ and SjcF1 appears to persist.

Septal junctions in filamentous cyanobacteria can be considered as functional analogs to metazoan gap junctions, as both of them mediate chemical communication between cells by simple diffusion 16, 32, 33, 34. Gap junction channels are composed of two connexons (one from each cell) that connect the cells across the intercellular space 35. Each connexon is made of six connexins, which are membrane proteins that constitute the only structural subunits of the channel. Hence, although SepJ and connexins are not related at the sequence level (and SepJ is larger and more complex than connexins), septal junctions and gap junctions may be considered as structural as well as functional analogs. Whether the putative SepJ tetramer detected in this work is present in one cell or, alternatively, dimers from adjacent cells form a tetramer is not yet known.

As a possible constituent of septal junctions traversing septal PG nanopores, SepJ could interact with PG. PG‐interacting proteins may exhibit PG‐binding activity 36, and our results have shown that SepJ, indeed, is able to bind PG. Further, the coiled‐coil domain of SepJ may be at least partially responsible for binding, as the SepJ‐CC‐His_6_ showed a binding activity close to that of the whole SepJ‐His_6_ protein. This observation strongly supports the SepJ topology in which the long N‐terminal extramembrane section is periplasmic. Another PG‐interacting protein involved in nanopore formation, SjcF1, bears recognizable PG‐binding domains 19. No amino acid sequence(s) homologous to known domains of interaction with PG (such as LytM domains) could, however, be found in SepJ. Instead, as predicted by Phyre2 37, the coiled‐coil domain of SepJ shows highest structural similarity to the coiled‐coil domain of PcsB from *Streptococcus pneumoniae*, a protein involved in PG hydrolysis 38. The periplasmic location of the N‐terminal extramembrane section of SepJ raises the question of the mechanism of export, which is unknown. The analysis of the purified SepJ‐GFP‐His_10_ protein showed the presence of two SepJ bands, suggesting that SepJ may be specifically processed to remove a short N‐terminal fragment. Whether such processing is related to the export of the long extramembrane domain of SepJ to the periplasm remains to be investigated.

## Conclusions

The results presented in this work provide information relevant for the understanding of the possible role of SepJ as a component of septal junctions in *Anabaena*. The SepJ protein can form multimers and binds to PG with a relevant role of the coiled‐coil domain in binding. These observations support the idea that SepJ‐related septal junctions contain SepJ complexes in which the extramembrane section of the protein resides in the periplasm, where the protein multimer could form a conduit communicating adjacent cells.

## Author contributions

FR‐L performed experiments; VM constructed strains; FR‐L, VM, and NB designed experiments; NB and E‐MA contributed analytic tools; EF conceived the study and wrote the manuscript; all authors analyzed results and made manuscript revisions.

## Supporting information


**Appendix S1.** Putative N‐terminal processing of SepJ.
