# Supplementary material for: Septal protein SepJ from the heterocyst‐forming cyanobacterium Anabaena forms multimers and interacts with peptidoglycan
Source: FEBS Open Bio. 2017 Aug 30;7(10):1515–26. doi: 10.1002/2211-5463.12280 (PMC5623728; doi:10.1002/2211-5463.12280)
Supplement: Supplementary file 1 — Appendix S1. Putative N‐terminal processing of SepJ. [file FEB4-7-1515-s001.pdf]

SUPPLEMENTARY MATERIAL

Septal protein SepJ from the heterocyst-forming cyanobacterium *Anabaena* forms multimers and interacts with peptidoglycan

Félix Ramos-León[1], Vicente Mariscal[1], Natalia Battchikova[2], Eva-Mari Aro[2], and Enrique Flores[1]

[1]Instituto de Bioquímica Vegetal y Fotosíntesis, CSIC and Universidad de Sevilla, Seville, Spain, and [2]Laboratory of Molecular Plant Biology, Department of Biochemistry, University of Turku, FI-20014, Turku, Finland.

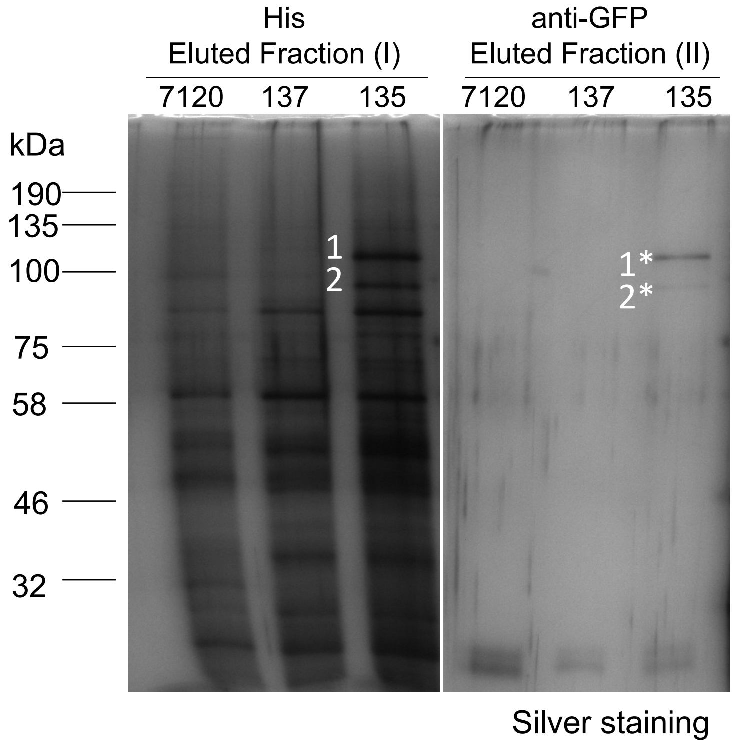

These gels are those shown in Fig. 3D of the article. The numbers inside the gels indicate the bands that were analyzed by mass spectrometry. Peptides identified corresponding to SepJ are shown in the next pages. Peptides corresponding to the GFP were also identified in all cases (not shown), corroborating that the bands contain the SepJ-GFP protein that is produced in strain CSVM135 (i.e., SepJ-GFP-His_{10} fusion protein).

Band 1

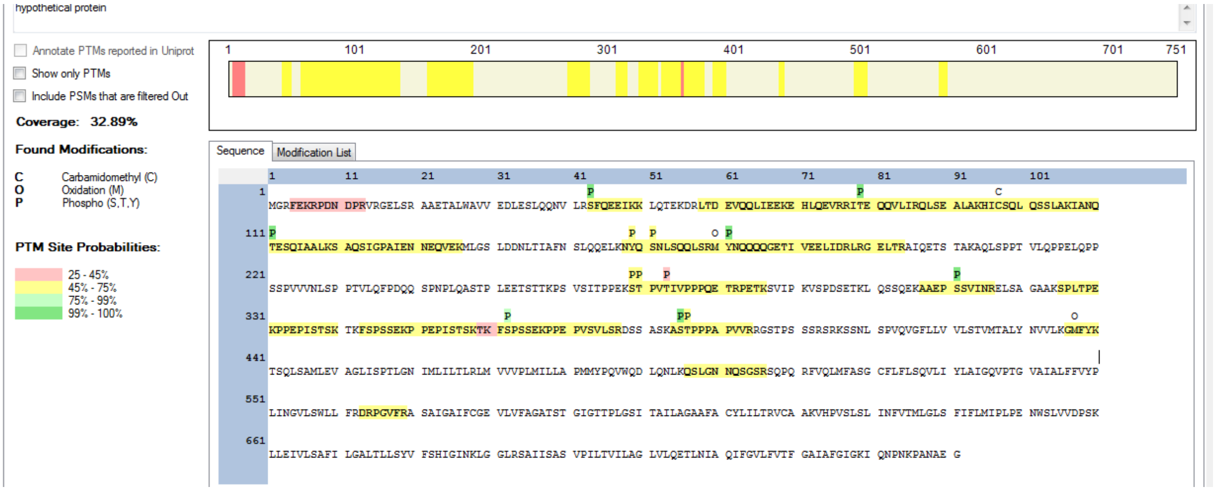

Band 1*

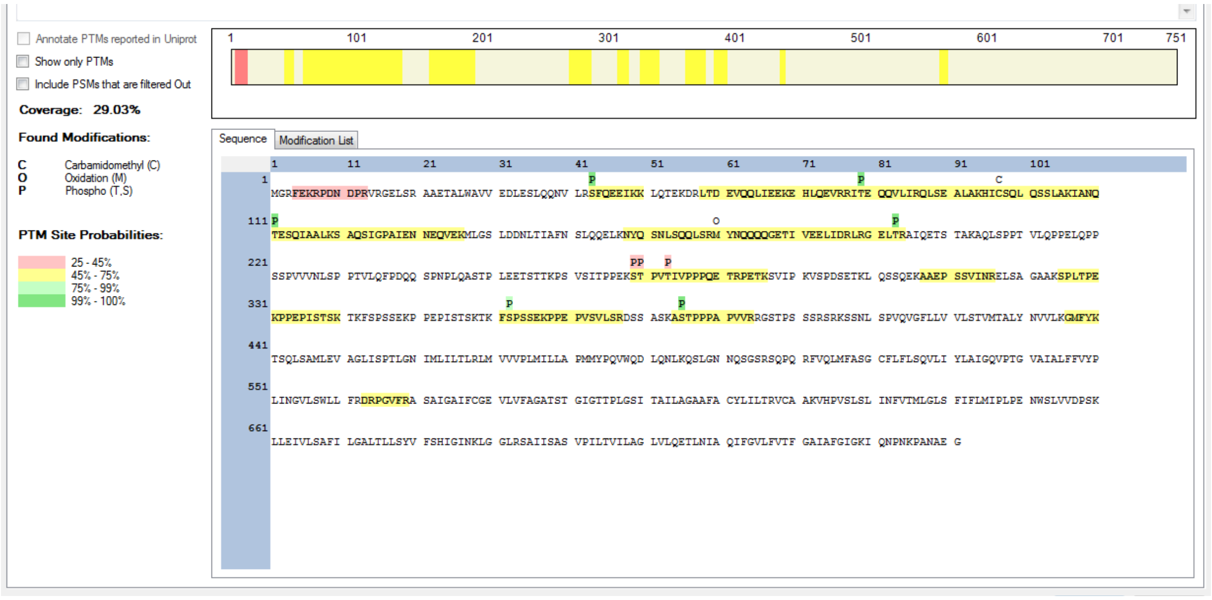

Note the detection of a peptide corresponding to amino acid residues 4 to 13 in bands 1 and 1*.

Band 2

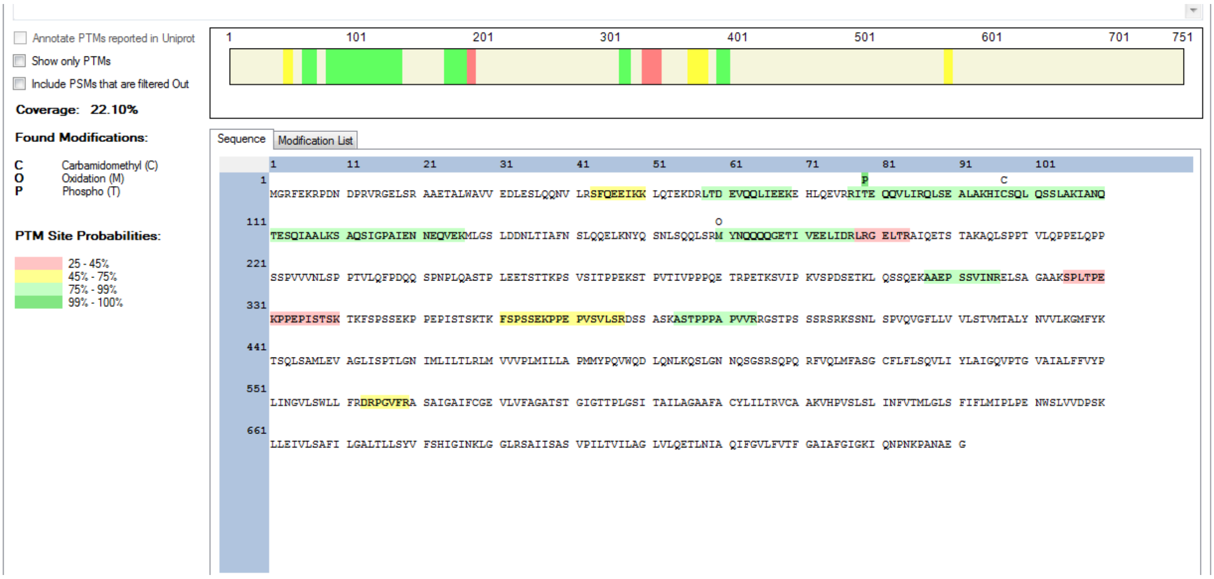

Band 2*

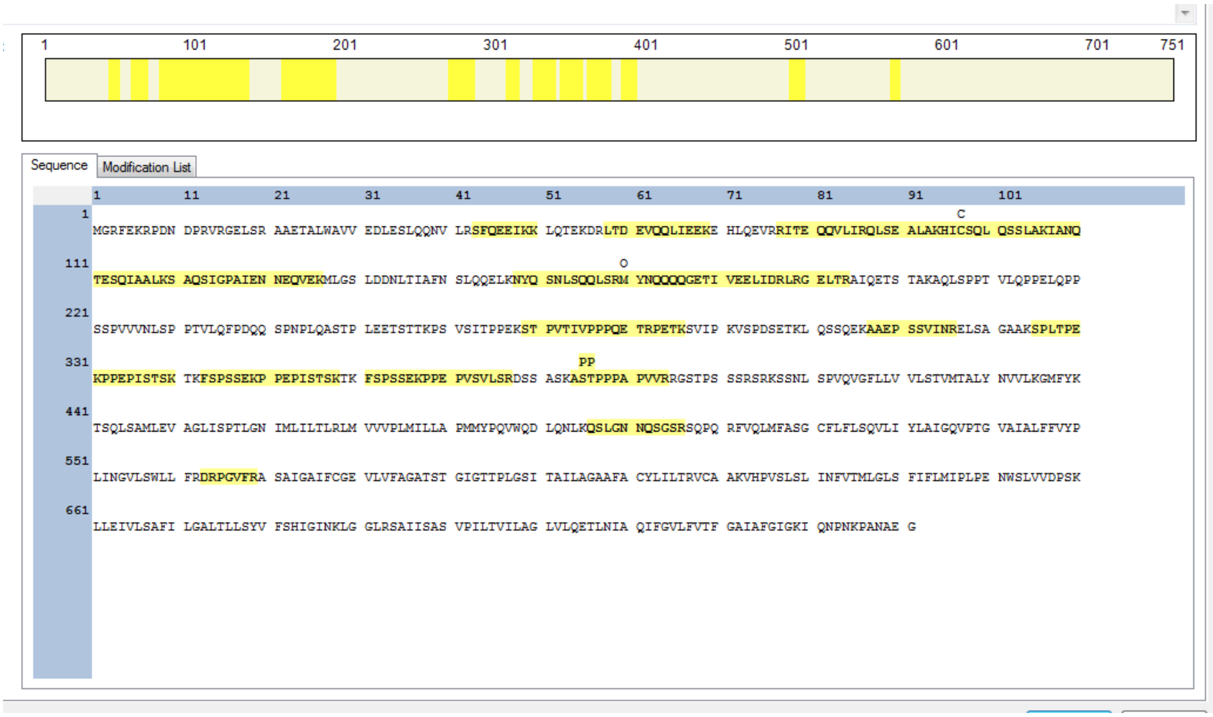

Note lack of detection of the peptide corresponding to amino acid residues 4 to 13 in bands 2 and 2*.
